# miR-452 Reverses Abnormal Glycosylation Modification of ERα and Estrogen Resistance in TNBC (Triple-Negative Breast Cancer) Through Targeting UGT1A1

**DOI:** 10.3389/fonc.2020.01509

**Published:** 2020-08-26

**Authors:** Yan Li, Yidong Zhou, Feng Mao, Songjie Shen, Bin Zhao, Yali Xu, Yan Lin, Xiaohui Zhang, Xi Cao, Ying Xu, Chang Chen, Jinqian Zhang, Qiang Sun

**Affiliations:** ^1^Department of Breast Surgery, Peking Union Medical College Hospital, Peking Union Medical College, Chinese Academy of Medical Sciences, Beijing, China; ^2^Department of Laboratory Medicine and Central Laboratories, Guangdong Second Provincial General Hospital, Guangzhou, China

**Keywords:** triple-negative breast cancer (TNBC), estrogen receptor (ER), uridine diphosphate glucuronyl transferase (UDPGT, UGT), glycosylation modification, estrogen resistance

## Abstract

**Background:** The breast epithelial cells in patients with triple-negative breast cancer (TNBC) actually have specific estrogen receptor (ER) expression, and the abnormal glycosylation of UGT1A1 in TNBC cells resulted in abnormal expression and function of ERα through regulating the modification of ERα. Therefore, our study targets the role of UGT1A1 expression, then glycosylation modification of ERα (estrogen receptor α) and estrogen resistance in development of TNBC.

**Methods:** The differential expression of mRNA and miRNA in TNBC tissues was tested. Luciferase activity was analyzed in TNBC cells treated with miR-452. Moreover, the human mammary gland and TNBC cell lines were dealt with estrogen and miR-452 or its inhibitors, then proliferation ability was further determined. Moreover, the role of interaction between UGT1A1 and ERα in the glycosylation modification of ERα and UGT activity, and metabolism of estrogen were assessed. The effects of miR-452 on TNBC by improving abnormal glycosylation modification of ERα by targeting UGT1A1 and estrogen resistance were studied *in vitro* and *in vivo*.

**Results:** The expression level of UGT1A1 in TNBC tumor tissues was higher than its matched para-tumorous tissues, but the miR-452 expression was opposite. The glycosylation modification site of ERα expressed in TNBC cells was different from that of normal mammary epithelial cells. The estrogen 17β-estradiol (E2) significantly promoted mitotic entry of TNBC cells. The interaction between UGT1A1 and ERα affected the expression level of each other, as well as the UGT enzyme activity and proliferation of TNBC cells. UGT1A1 induced production of intracellular estrogens and TNBC proliferation, but it could be reversed by overexpression of ERα. Upregulation of ERα caused the downregulation of UGT1A1 and marked decrease of intracellular estrogen products, and then suppressed TNBC proliferation. Moreover, UGT1A1 was the target gene of miR-452; miR-452 antagomir restrained TNBC xenograft.

**Conclusion:** Our results demonstrated that estrogen was a positive factor in the proliferation of TNBC cells at onset of mitosis through accentuating the expression and enzyme activity of UGT1A1. However, miR-452 targeted to UGT1A1, then regulated glycosylation modification of ERα, estrogen metabolism, and TNBC development associated with estrogen resistance.

## Introduction

Breast cancer (BC) is the woman's malignant tumor with the highest incidence and the second highest mortality rate in the world (1). Recently, the incidence of BC has increased rapidly; BC seriously threatens people's health and causes huge social and economic burdens (1). The most dangerous type in BC is TNBC (triple-negative breast cancer). It is highly invasive, easy to metastasize, and has a poor prognosis (2). Moreover, lack of HER2 (human epidermal growth factor receptor-2), PR (progesterone receptor), and ER (estrogen receptor) expressions are present in TNBC. Therefore, traditional local treatments such as chemotherapy, radiotherapy, and surgery that are currently the main systemic therapies, and even HER2 targeted therapy all have little benefit to TNBC. In addition, owing to the existence of multiple variant loci of ER, PR, or HER2 in TNBC subtypes, even in the same treatment regimen in different patients will receive different responses that are very clear (3). Because the exact etiology and pathogenesis of TNBC is unknown, new targeted drugs have not achieved satisfactory clinical results (3). Therefore, to clarify the pathogenesis of TNBC, exploring effective therapeutic drugs and methods have been the key issues in the field of prevention and therapy for TNBC.

Data from current clinical studies confirm that the incidence of BC is positively correlated with estrogen levels of patients (4). Traditionally, the BC cells in TNBC patients do not express ER. However, recent studies have found that breast epithelial cells in patients with TNBC actually have specific ER expression, although it is a low expression level (5). The results of our previous work also were coincident with their discovery. This has made it possible to study the genesis and development mechanism of TNBC associated with the estrogen metabolism pathways.

Uridine diphosphate glucuronyl transferase (UDPGT, UGT) is one of the vital factors for combination and then removing endogenous compounds or toxic xenobiotics (1, 6). UGTs are able to catalyze umbelliferone, 17alpha-ethinylestradiol, and 17beta-estradiol (1, 7). One member of this family, as well as a UDP-glucuronosyltransferase UGT1A1, can glucuronidate hormones, steroids, and excretable metabolites (7–13).

Our previous research results suggested that (1) UGT expression level was related to treatment strategy and prognosis of TNBC, (2) miR-452 regulated UGT1A1 expression in TNBC cells, and (3) the abnormal expression and enzyme activity of UGT1A1 in TNBC cells resulted in abnormal expression and function of ERα through regulating the modification of ERα. Consequently, this abnormal status of ERα further led to estrogen metabolic disorders and estrogen resistance in TNBC cells, then affected the biological behavior of TNBC cells. Therefore, we proposed the hypothesis that miR-452 may reverse the abnormal glycosylation of ERα in TNBC cells by regulating the expression and function of UGT1A1, then improved the estrogen metabolism disorder and estrogen resistance in TNBC cells. Finally, the inhibition of occurrence and development in TNBC were purposefully achieved.

To verify the aforementioned hypothesis, this project aimed to explore the pivotal mechanism of the occurrence and development of TNBC by targeting estrogen metabolism and estrogen resistance associated with ERα expression and its abnormal glycosylation in TNBC. Moreover, it was planned to explore how miR-452 affected the estrogen metabolism in TNBC cells through regulating UGT1A1 expression.

## Materials and Methods

### Samples

After mastectomy, tissues (non-tumor, para-tumor, and tumor) of each patient were obtained at our department (Department of Breast Surgery, Peking Union Medical College Hospital) from January 2010 to December 2018. The utilization of these specimens in our study has been approved by the Ethics Committee of Peking Union Medical College Hospital, Peking Union Medical College (Beijing, China) based on the Declaration of Helsinki. All informed consents were signed by every participant patient.

### Assessment of Differential mRNA Expression

Total RNAs were extracted from tissues using TRIzol, and after performance of reverse transcription, cDNAs were obtained. Subsequently, Agilent 8 × 60 K gene chip was used to detect gene expression profiles in tissues. Finally, differential analysis was conducted according to traditional methods (14–17). DESeq2 was used to analyze these data with online software Insilicom BioKDE (insilicom.com) (18–21).

### Western Blot and CO-IP (Co-immunoprecipitation) Analysis

The proteins were dealt with SDS-PAGE (sodium dodecyl sulfate–polyacrylamide gel electrophoresis) gel, then transferring of membranes was performed. Moreover, membranes were blocked with skim milk and probed using the primary antibody. The following antibodies were used in this study: β-actin (Abcam; ab194697: WB 1:2000), UGT1A1 (Abcam; ab194697: WB 1:2000; IF 1:400), and anti-Estrogen Receptor alpha (ERα) antibody (Abcam; ab32063: WB 1:1000; IF 1:200), respectively. Then the chemiluminescence-based detection was conducted according to the secondary antibody conjugated horseradish peroxidase with exposed film. The assays of co-immunoprecipitation (CO-IP) were immunoprecipitated with Suspension Agarose beads (Protein G Plus/Protein A).

### Immunohistochemistry Test

Tissues were fixed with neutral formalin (10%), then dehydrated and embedded with paraffin. Moreover, sections (5 μm) were cut from these tissues; primary antibody of UGT1A1 (Abcam, ab194697, 1:200) and the matched secondary antibody were further used to incubate after antigen retrieval, respectively. Besides, after staining with DAB substrate kit (Pierce, USA), these sections were observed under Olympus microscope to obtain a photograph of the expression status, including subcellular location, expression level, and expression density.

### Differential miRNA Analysis

After miRNA extraction from the aforementioned total RNAs using miRNeasy Mini Kit, next-generation sequencing was performed with Illumina HiSeq 2000 platform. Then miRBase (version 19.0) was matched to the mature sequences of miRNAs based on reference sequences (22, 23) and reference library of Bowtie (version 0.12.7) (24). Finally, these data were normalized according to reads per million (25).

### Cell Lines

The human mammary gland cell line of epithelial spontaneous immortalization MCF-12A (ATCC CRL-10782), human BC cell line MCF-7 (ATCC HTB-22), and TNBC cell line MDA-MB-231 (ATCC HTB-26) were all purchased from American Type Culture Collection (ATCC) and cultured based on the manufacturer instructions. MDA-MB-231 was dealt with 17β-estradiol (E2) purchased from Santa Cruz Biotechnology (Santa Cruz, CA, USA; CAS 50-28-2).

### qRT-PCR

TaqMan miRNA test was conducted to assess the miR-452 expression level with SYBR Green kit. The primer sequences are shown as follows: miR-452: 5′-AACUGUUUGCAGAGGAAACUG-3′ (upstream) and 5′-GUUUCCUCUCUGCAAACAGUUUU-3′ (downstream); U6: 5′-TGACCTGAAACATACACGGGA-3′ (upstream) and 5′-TATCGTTGTACTCCACTCCTTGAC-3′ (downstream); UGT1A1: 5′-TTGGGAGTGCGGGATTCAAA-3′ (upstream) and 5′-CTGGGGTTATTTCTGGGCGA-3′ (downstream); ERα: 5′-AGCTGGGCCAAGAAGATTCC-3′ (upstream) and 5′-GAGCAGATGTTCCATGCCCT-3′ (downstream); GAPDH (glyceraldehyde-3-phosphate dehydrogenase): 5′-CTCATGACCACAGTCCATGCC-3′ (upstream) and 5′-GGCATGGACTGTGGTCATGAG-3′ (downstream).

### Reporter Gene Analysis

UGT1A1 was predicted as the target gene of miR-452 with Targetscan (version 7.2), an online software. The further identified reporter gene assay was conducted, and recombinant pmirGLO-UGT1A1 was constructed based on 3′UTR sequence of its mRNAs. The sequence was AGCUGGAGCAUGUU(CAGAUGA)G (Table 1), its position located at 17–23 matching to GUGAAUGAAGAAAC(GUCUACU)C of seed sequence of miR-452. The recombinant mutated pmirGLO-UGT1A1 was also constructed with Site-Mutation kit. The luciferase activity was assessed with Dual-Glo Luciferase Assay System.

**Table 1:** The predicted consequential pairing site between ERα and miR-452.

	**Predicted consequential pairing**	**Site type**	**Context++ score**	**Context++ score**	**Weighted context++**	**Conserved branch**	**P_**CT**_**
	**of target region (top)**			**percentile**	**score**	**length**	
	**and miRNA (bottom)**						
Position 17–23 of	5′.GAGCUGGAGCAUGUUCAGAUGAG.	7mer-m8	−0.06	67	−0.06	0	N/A
UGT1A1 3′ UTR							
hsa-miR-452-3p	|||||||						
	3′ GUGAAUGAAGAAACGUCUACUC						

### Mimics and Inhibitors of miR-452

The mimics and inhibitors of miR-452 were transfected into cells with Lipofectamine 3000. The sequences are shown as follows: mimics: 5′-UGAAACAUACACGGGAAACCUC GCGAACUGUUUGCAGAGG-3′ (sense) and 5′-CAGUGCGUGUCGUGGAGU-3′ (antisense); mimics control: sense: 5′-gucguauccagugcgugucguggagucggcaauugcacuggauacgacucaguuu-3′ and antisense: 5′-aaacugagucguauccagugcaauugccgacuccacgacacgcacuggauacgac-3′; antagomir: 5′-GAGGUUUCCCGUGUAUGUUUCA-3′; NC (negative control): sense: 5′-ACGUGACACGUUCGGAGAAUU-3′ and antisense: 5′-AAUUCUCCGAACGUGUCACGU-3′; inhibitors: 5′-AACUGUUUGCAGAGGAAACUGA-3′; inhibitor control: GGUUCGUACGUACACUGUUCA.

### Adenovirus Vector

Ad-UGT1A1 and Ad-ERα, the recombinant adenovirus vectors, were produced using AdEasy (Ad) Vector System (Stratagene, La Jolla, CA, USA). The infection controls were Ad-GFP.

Adenovirus-bacterial recombination system (AdEasy) was used, including pGEM-3ZF (+) and pAdEasy-1 pShuttle-SYN, which were all expression vectors labeled using GFP (green fluorescent protein). The total RNA extracted from cells or tumor tissues of patients with TNBC was used for gene amplification of UGT1A1, and then it was cloned into vectors based on human UGT1A1 (NM_000463.3) sequence in GenBank. Moreover, the sequence of primer at upward was 5′-GAATTCATGGCTGTGGAGTCCCAGGG-3′ and the sequence of primer at downward was 5′-AAGCTTTCAATGGGTCTTGGATTTGTGGG-3′.

The total RNA extracted from human mammary gland cell line of epithelial spontaneous immortalization MCF-12A was used for gene amplification of ERα, and then it was cloned into vectors based on human ERα (NM_000125.4) sequence in GenBank. Moreover, the sequence of primer at upward was 5′-GAATTCATGACCATGACCCTCCACACCA-3′ and the sequence of primer at downward was 5′-AAGCTTGACCGTGGCAGGGAAACCCT-3′.

The evaluation for concentration of virus and the titer testing of viral vectors were performed, and then viral stocks were detected on the replication competent viruses with potential contamination. The concentrations of virus were measured at A_260_. The titers of viral vectors Ad-UGT1A1, Ad-ERα, and Ad-GFP were 3.2 × 10^12^, 2.3 × 10^12^, and 2.6 × 10^12^ pfu/ml, respectively.

### Cell Transfection of siRNA

The target sequence of UGT1A1 siRNA is CCCACTTACTGCACAACAA. The siRNA of scrambled sequence and UGT1A1 siRNA sequence are indicated as follows: scrambled siRNA: 5′-ACUUUGCUGUAACCCUGUAdTdT-3′ (sense), 5′-UACAGGGUUACAGCAAAGUdTdT-3′ (antisense); UGT1A1 siRNA: 5′-CCCACUUACUGCACAACAA dTdT-3′ (sense), 5′-dTdT GGGUGAAUGACGUGUUGUU-3′ (antisense). The siRNA of ERα (sc-29305) was purchased from Santa Cruz Biotechnology (Dallas, Texas, USA).

### The Activity Analysis of UGT

The UGT-Glo assay (Promega) was used to detect the activity of UGT. At first, after freeze–thawed three times, and the homogenates of cells with glass homogenizer were prepared. Second, BCA method was used to examine the protein concentration of homogenates, which was subsequently incubated with alamethicin, UGT Multienzyme Substrate (50 μM), and buffer of UGT-Glo. Furthermore, final concentration of UDPGA (4 mM) was used for reactions, after that the stabilization of reactions was conducted with D-Cysteine + Luciferin Detection Reagent. Finally, the Turner Biosystems luminometer was used to read the values (26).

### The Synchronization of Cells and Labeling With BrdU for Detection of Mitotic Index

At first, cell synchronization was performed with block method using double thymidine, which was the reversible arresting agent for cell cycle. After that, cells were arrested at the early *S* phase. Besides, the M-phase arrest cells were blocked with nocodazole (Sigma) at early *S* phase. Subsequently, the synthesis of DNA was evaluated using label of BrdU, and the positive cells stained with antibody of anti-BrdU were counted manually using immunofluorescence microscope. Moreover, mitotic events were also scored based on the staining of DNA using DNA dye (Hoechst 33258) and time-lapse videomicroscopy (captured with Openlab software). The morphological changes of cells were also observed, including the nucleus morphology and DNA condensation. Furthermore, the analysis of flow cytometric after staining phospho-H3 was used to assess the mitotic index.

### Determination of Estrogen Products With HPLC-RIA

The estrogen levels including estrone sulfate, estradiol, and estrone were simultaneously detected using the high-performance liquid chromatography–radioimmunoassay (HPLC-RIA) method with radiolabeled estrogens (26). Estradiol-6-(O-carboxymethyl)-oximino-2-(2-[^125^I]-iodo-histamine) (2000 Ci/mmol) was purchased from Amersham International (UK); [2,4,6,7,16,17-3H]E2 (170 Ci/mmol), [6,7,-3H]E1S (60 Ci/mmol), and [2,4,6,7,-3H]E1 (101 Ci/mmol) were obtained from DuPont NEN (Boston, MA, USA), respectively. First, the estrogens were extracted from cells using chromatography with Lipidex-5000 (27). Second, individual estrogens were separated with a HPLC system (28, 29). The measurements of E1 (estrone), E2 (estradiol), and E1S (estrone sulfate) were simultaneously conducted by RIA (30–32), then the values of three main fractions of estrogen were finally obtained.

### Statistical Analysis

All data were represented with mean ± *SD*. Statistical analysis was conducted with GraphPad Prism (version 8.0). Between the two groups, Student's *t*-test was used to analyze their differences. ANOVA with Kruskal–Wallis *post-hoc* tests was used for multiple group comparisons. *P* < 0.05 was statistical difference with significance.

## Results

### Differential Expression Analysis of mRNA Profiling

The differential mRNA expression profiles were assessed between tumorous and para-tumorous tissues using gene chip. Therefore, our results indicated 21 differential mRNAs, including three downregulated mRNAs and 18 upregulated mRNAs in TNBC tumor tissues (Table 2). Moreover, it indicated overexpression of UGT1A1 in TNBC tumor tissues compared with para-tumor tissues.

**Table 2:** Significantly differentially expressed (fold change ≥ 2 and adjusted *p* ≤ 0.05) genes in various comparisons.

**mRNA**	**Fold-change (tumor/para-tumorous tissues)**	***t-*value**	***P*-value**
RecD–like DNA helicase YrrC	7.62	7.272	8.63 E-04
ATP-dependent DNA ligase	6.41	8.239	3.96 E-03
Lysine decarboxylase family	5.73	5.824	8.42 E-03
Deoxyadenosine kinase (gp051)	5.39	8.118	6.91 E-03
**Uridine diphosphate glucuronyl transferase 1 (UDPGT1, UGT1)**	5.02	8.181	6.57 E-03
NrdR–regulated deoxyribonucleotide transporter	4.27	3.691	5.85 E-03
Glutaredoxin	4.14	6.133	2.68 E-03
ATP/GTP binding protein	3.45	4.712	4.53 E-03
Replicative DNA helicase (DnaB)	3.36	5.145	6.79 E-03
Phage toprim domain containing protein	2.67	2.104	5.33 E-03
Single–stranded–DNA–specific exonuclease RecJ	2.52	6.613	7.63 E-03
Thymidylate synthase partial	2.48	8.215	4.73 E-03
Deoxyuridine 5'–triphosphate nucleotidohydrolase	2.47	3.132	6.45 E-03
Ribonuclease HI	2.37	4.274	7.36 E-03
Beta glucosyl transferase	2.26	3.482	8.56 E-04
DNA polymerase III alpha subunit	2.23	4.269	4.38 E-03
Guanylate kinase/L-type calcium channel region	2.17	2.832	2.04 E-03
rusA family endodeoxyribonuclease	2.09	5.618	7.34 E-03
Topoisomerase IV subunit B	0.35	−0.362	6.27 E-03
Aggregation promoting factor	0.33	−0.215	5.31 E-03
Phage protein EFP_gp137	0.18	−0.153	3.56 E-03

*The meaning of the bold font is the research target in this paper*.

### High Expression of UGT1A1 in TNBC

**Figure 1:**
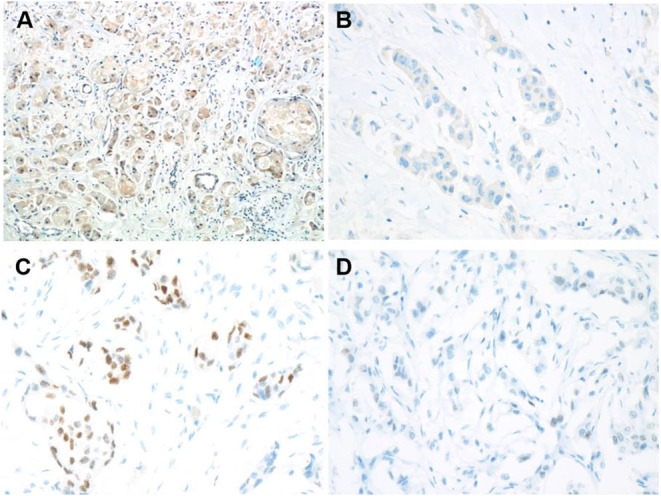
Low expression of ERα and high expression of UGT1A1 in TNBC. To explore the expression of UGT1A1 in TNBC, immunohistochemistry staining was conducted. The results indicated a high expression level of UGT1A1 in tumorous tissues of patients with TNBC **(A)**. We found that UGT1A1 was present in the nucleus, cytoplasm, and cell surface of para-tumorous tissues at a low expression level **(B)**. The immunohistochemical staining was used to conduct the determination of ERα expression in TNBC tissues and their paired normal control tissues. Consequently, the positive stains of ERα were observed in the normal mammary gland tissues with the moderate level **(C)**. However, in TNBC tissues, the low expression of ERα was visible in endothelium **(D)**.

**Figure 2:**
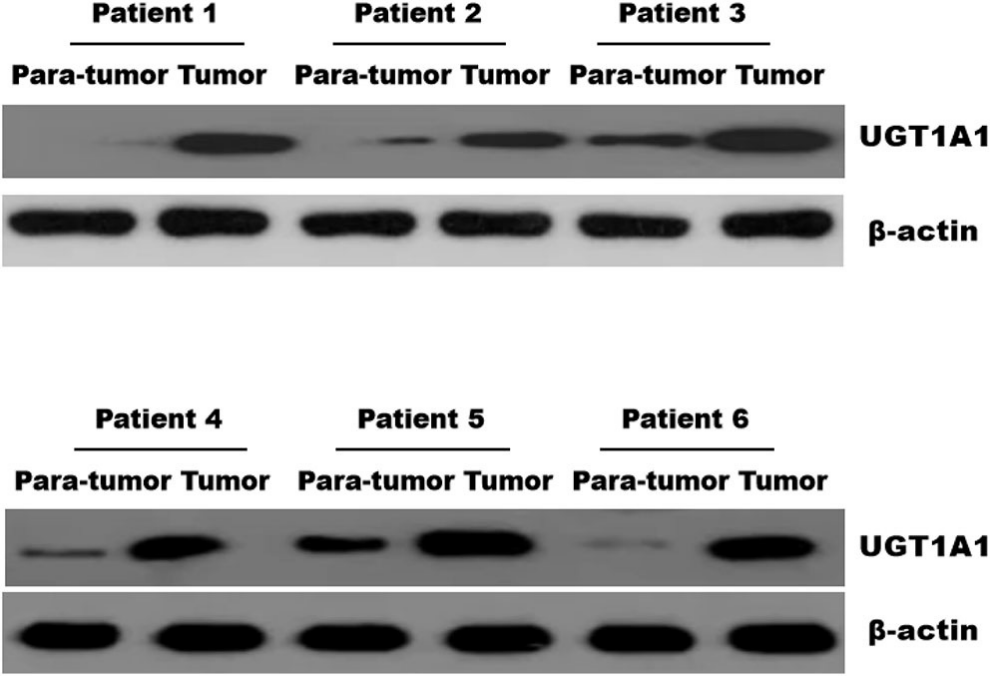
The expression of UGT1A1 in tissues of patients with TNBC. Moreover, the expression of UGT1A1 in tumorous tissues of patients with TNBC was much more than in the matched para-tumorous tissues.

Immunohistochemistry staining was then conducted to explore UGT1A1 expression in TNBC. The results indicated high level of UGT1A1 expression in tumor tissue of patient with TNBC (Figure 1A). We found that UGT1A1 was expressed in the nucleus, cytoplasm, and cell surface in para-tumor tissues at a low expression level (Figure 1B). However, the expression of UGT1A1 in tissues of patients with TNBC was much more than the matched para-tumor tissues (Figure 2).

### Low Expression of ERα in TNBC

In fact, it was well-known that TNBC tissues do not express ERα. However, our results demonstrated low expression ERα level with abnormal state in TNBC patients (Figure 1D). Moreover, ERα is present in the nucleus, cytoplasm, and cell surface of normal mammary glands at a moderate expression level (Figure 1C). This also laid the foundation for the study of ERα as a novel target for treatment of TNBC.

### UGT1A1 Induced Glycosylation Modification of ERα

**Figure 3:**
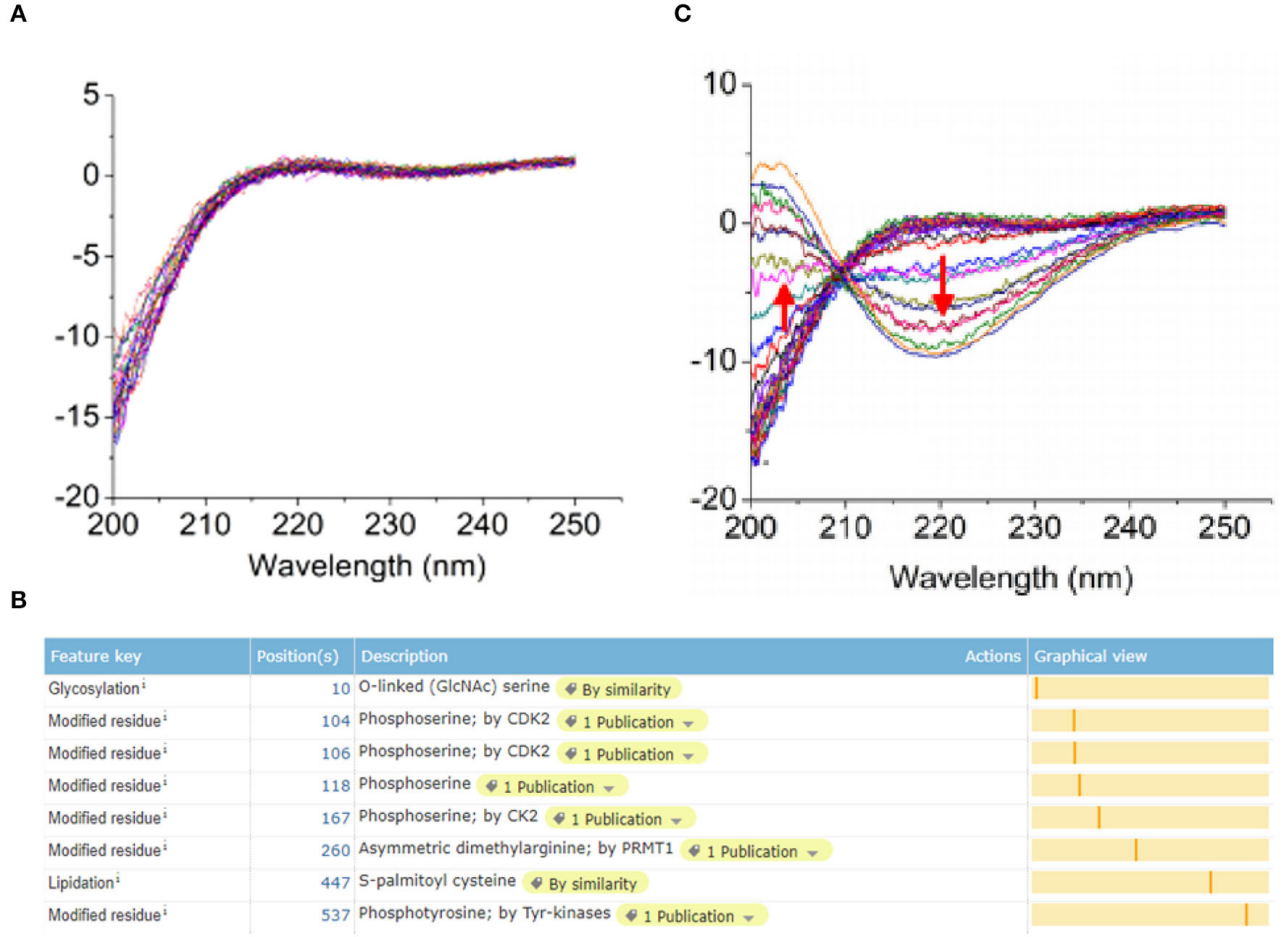
Post-translational glycosylation modification of ERα induced by UGT1A1. The ERα protein expression in normal human breast epithelial cells has a modification site at the 10th amino acid with the O-GalNAc glycosylation **(A)**, which was in accord with the bio-information analysis based on the online software of Uniprot (https://www.uniprot.org/) **(B)**. However, the expression of ERα protein was present on the surface of TNBC cells with a low expression level, whereas the glycosylation site at the 10th amino acid site was UDP-GlcNAc glycosylation modification **(C)**.

ERα protein expressed in breast epithelial cells of normal human has a modification site at the 10th amino acid with the O-GalNAc glycosylation (Figure 3A), which was in accord with the bio-information analysis based on the online software of Uniprot (https://www.uniprot.org/) (Figure 3B). However, the protein expression of ERα on the surface of TNBC cells has a low expression level, whereas the glycosylation site at the 10th amino acid site was UDP-GlcNAc glycosylation modification (Figure 3C).

### Estrogen Upregulated the Expression of UGT1A1 and Its Enzyme Activity in MDA-MB-231 While Promoting Its Proliferation

**Figure 4:**
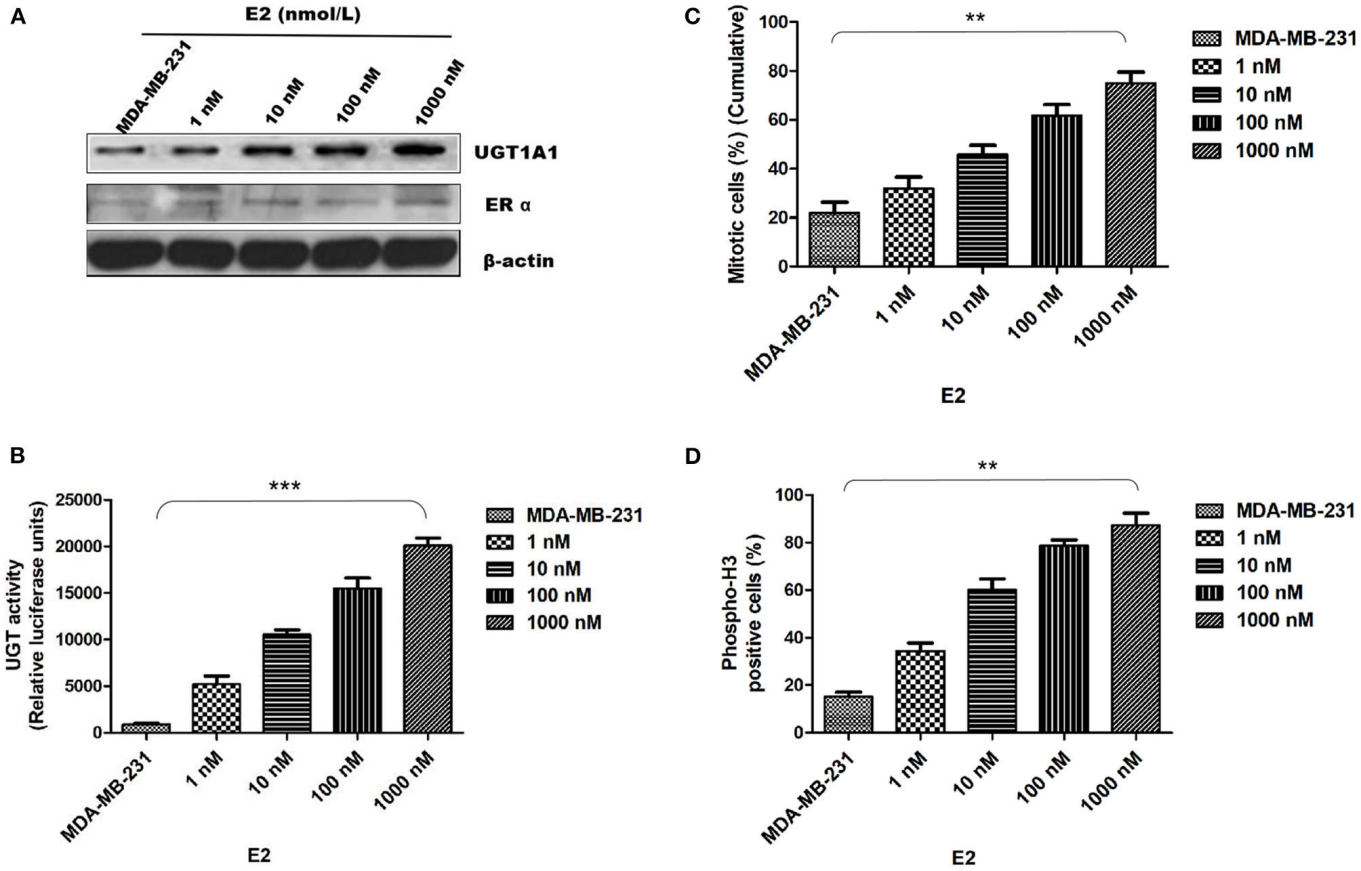
Estrogen upregulated the expression of UGT1A1 and its enzyme activity in TNBC cell line MDA-MB-231, and promoted cell proliferation. In this experiment, the cell line MDA-MB-231 was treated with 17β-estradiol (E2) in the presence of concentration gradients. Our results demonstrated that the 17β-estradiol (E2) could strongly promote the expression of UGT1A1 in a dose-dependent manner, but lightly upregulate the ERα expression **(A)**. Moreover, the enzyme activity of UGT1A1 was determined, and these results indicated the presence of concentration gradients, too. Our results demonstrated that the E2 induced forcefully the UGT1A1 activity in a dose-dependent manner **(B)**. Furthermore, the cell mitosis was released after using the block with double thymidine to synchronize cell at the G1/S phase, then the synthesis of DNA was evaluated with BrdU. Incorporating BrdU into the control, the mitotic cells treated with E2 increased obviously than control in a dose-dependent manner **(C)**. Our results indicated that the expression and enzyme activity of UGT1A1 upregulated by E2 may control the DNA synthesis, and then transition of G1/S boundary or mitosis in TNBC cells. The flow cytometry and stain of phospho-H3 was additionally used to confirm the role of UGT1A1 expression and enzyme activity in mitotic entry of TNBC cells **(D)**. Estrogen 17β-estradiol (E2) significantly promoted mitotic entry of TNBC cells. In summary, these results sustained powerfully the estrogen as a positive factor in the proliferation of TNBC cells at onset of mitosis through accentuating the expression and enzyme activity of UGT1A1. **P < 0.01, ***P < 0.001.

In this experiment, the human TNBC cell line MDA-MB-231 was dealt with 17β-estradiol (E2) in the presence of concentration gradients (Figure 4). Our results demonstrated that the 17β-estradiol (E2) could strongly promote the expression of UGT1A1 in a dose-dependent manner, but lightly upregulate the ERα expression (Figure 4A). Moreover, the enzyme activity of UGT1A1 was determined, and these results indicated the presence of concentration gradients, too (Figure 4). Our results demonstrated that the E2 induced forcefully the UGT1A1 activity in a dose-dependent manner (Figure 4B). Furthermore, the cell mitosis was released after using the block with double thymidine to synchronize the cell at the G1/S phase, then DNA synthesis was evaluated using BrdU. Incorporating BrdU into the control, the mitotic cells treated with E2 increased more obviously than control and were dose dependent (Figure 4C). These results indicated that the expression and enzyme activity of UGT1A1 upregulated by E2 may control the DNA synthesis, and then transition of G1/S boundary or mitosis in TNBC cells. The flow cytometry and stain of phospho-H3 was additionally used to confirm the role of UGT1A1 expression and enzyme activity in mitotic entry of TNBC cells (Figure 4D). Estrogen 17β-estradiol (E2) significantly promoted mitotic entry of TNBC cells. In summary, these results sustained powerfully the estrogen as a positive factor in the proliferation of TNBC cells at onset of mitosis through accentuating the expression and enzyme activity of UGT1A1.

### The Interaction of UGT1A1 With ERα

**Figure 5:**
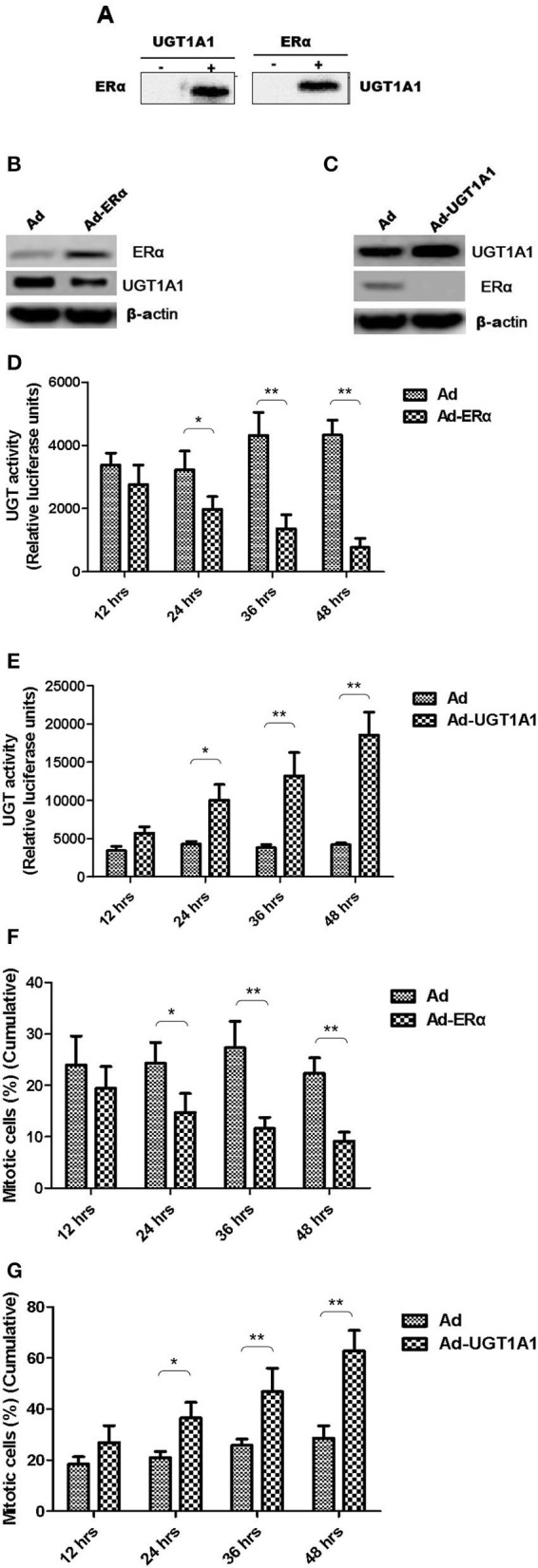
The interaction between UGT1A1 and ERα in TNBC cells. Moreover, the CO-IP assay was conducted to verify the interaction between UGT1A1 and ERα in TNBC cells **(A)**. Furthermore, upregulating expression of ERα induced remarkably the reduction of UGT1A1 expression **(B)**, and then the UGT enzyme activity also notably decreased in a time-dependent manner **(D)**. At the same time, overexpression of ERα inhibited dramatically the proliferation of MDA-MB-231 cells in a time-dependent manner **(F)**. Besides, upregulating expression of UGT1A1 caused prominently the decrease of ERα expression **(C)**, and the UGT enzyme activity significantly increased in a time-dependent manner, too **(E)**. Moreover, overexpression of UGT1A1 promoted markedly the proliferation of MDA-MB-231 cells in a time-dependent manner **(G)**. *P < 0.05, **P < 0.01.

Moreover, the CO-IP assay was conducted to verify the interaction between UGT1A1 and ERα in TNBC cells. Our results identified the intracellular interaction between UGT1A1 and ERα (Figure 5A). Furthermore, after upregulating the expression of ERα for 48 h, it induced remarkably the reduction of UGT1A1 expression (Figure 5B), and then the UGT enzyme activity also notably decreased in a time-dependent manner (Figure 5D). At the same time, overexpression of ERα inhibited dramatically the cell proliferation of MDA-MB-231 in a time-dependent manner (Figure 5F). Besides, upregulating expression of UGT1A1 for 48 h caused prominently the decrease of ERα expression (Figure 5C), and the UGT enzyme activity significantly increased in a time-dependent manner, too (Figure 5E). Moreover, overexpression of UGT1A1 promoted markedly the cell proliferation of MDA-MB-231 in a time-dependent manner (Figure 5G). These results confirmed that the interaction between UGT1A1 and ERα affected the expression level of each other as well as the UGT enzyme activity and proliferation of TNBC cells.

### UGT1A1 Induced Production of Intracellular Estrogens

**Figure 6:**
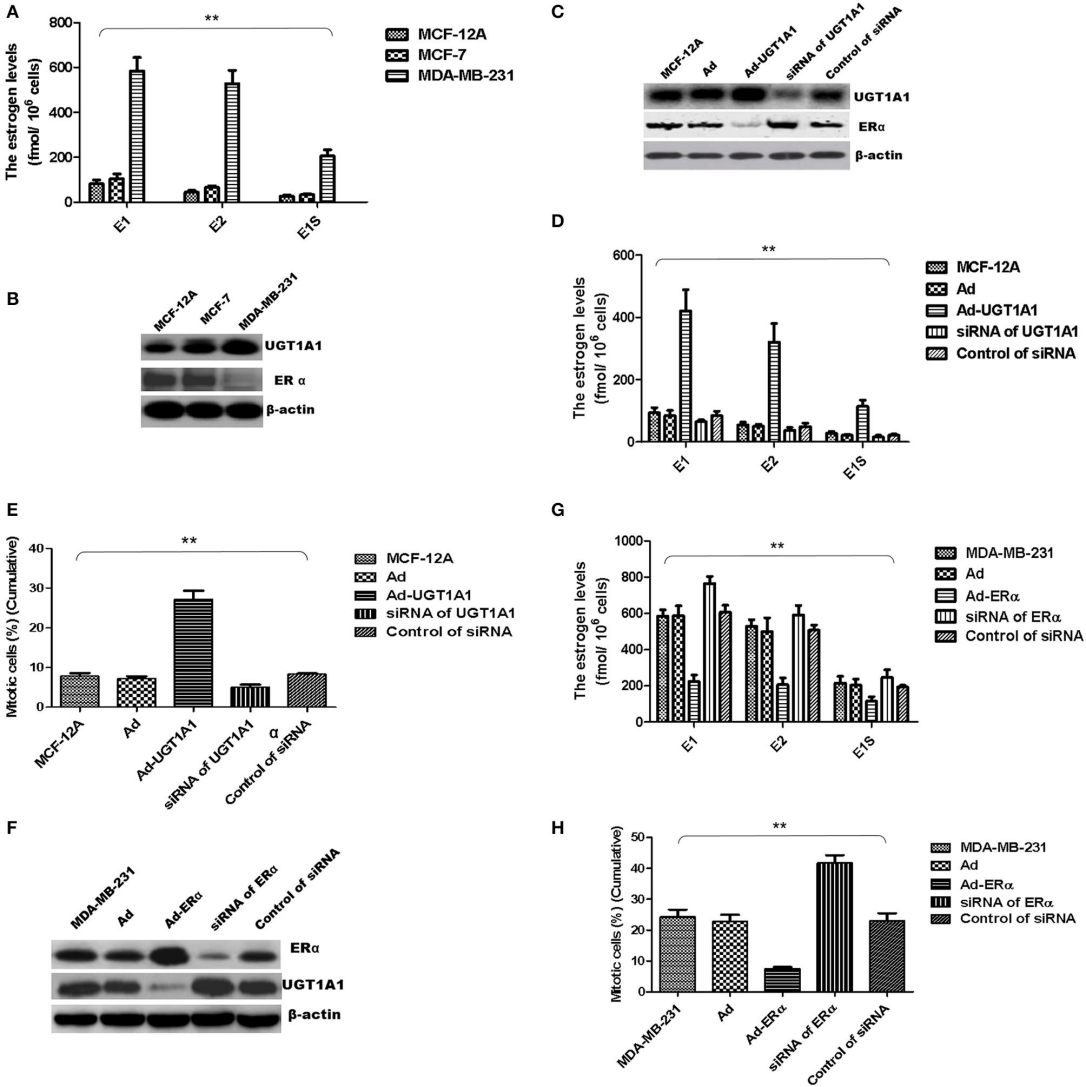
UGT1A1 induced production of intracellular estrogens. At first, the intracellular estrogen products in human mammary gland cell line MCF-12A, human breast cancer cell line MCF-7, and triple-negative breast cancer (TNBC) subtype cell line MDA-MB-231 were determined, respectively. In MDA-MB-231 cells, the productions of E1, E2, and E1S were all remarkably greater than that in MCF-12A and MCF-7. However, there was no significant difference between MCF-12A and MCF-7 **(A)**. Moreover, the expression levels of ERα and UGT1A1 were detected in these cell lines **(B)**. The results showed that the expression of UGT1A1 in MDA-MB-231 was much greater than MCF-12A and MCF-7, but the ERα expression in MDA-MB-231 was lower than MCF-12A and MCF-7. Meanwhile, the expression of UGT1A1 in MCF-7 was slightly more than MCF-12A, but there was no significant difference. Besides, the expression level of ERα in MCF-7 was slightly lower than MCF-12A, but there was no significant difference **(B)**. In cell line MCF-12A, overexpression of UGT1A1 downregulated the expression of ERα **(C)**, and then resulted in an obvious increase of intracellular estrogen products **(D)**. Meanwhile, Ad-UGT1A1 promoted markedly the cell proliferation of MCF-12A **(E)**. Besides, overexpression of ERα was induced in cell line MDA-MB-231, which caused the downregulation of UGT1A1 **(F)** and marked decrease of intracellular estrogen products **(G)**. Meanwhile, Ad-ERα significantly reduced cell proliferation of MDA-MB-231 **(H)**. **P < 0.01.

At first, the intracellular estrogen products in human mammary gland cell line of epithelial spontaneous immortalization MCF-12A, human BC cell line MCF-7, and TNBC cell line MDA-MB-231 were determined based on the aforementioned method, respectively (26). The products of E1, E2, and E1S in MDA-MB-231 were all remarkably greater than that in MCF-7 and MCF-12A. However, there was no significant difference between MCF-7 and MCF-12A (Figure 6A).

Moreover, the expression levels of ERα and UGT1A1 were detected in these cell lines (Figure 6B). In MDA-MB-231, UGT1A1 expression was much more than MCF-7 and MCF-12A, but the ERα expression was lower than that in MCF-12A and MCF-7. Meanwhile, the expression of UGT1A1 in MCF-7 was slightly more than MCF-12A, but no significant difference was found between MCF-7 and MCF-12A. Besides, the expression level of ERα in MCF-7 was slightly lower than MCF-12A, but there was no significant difference (Figure 6B).

In cell line MCF-12A, overexpression of UGT1A1 downregulated the expression of ERα (Figure 6C), and then resulted in an obvious increase of intracellular estrogen products (Figure 6D). It meant the suppression of estrogen metabolism, utilization of estrogens, and estrogen resistance. Meanwhile, Ad-UGT1A1 promoted markedly the cell proliferation of MCF-12A (Figure 6E).

Besides, overexpression of ERα was induced in cell line MDA-MB-231, which caused the downregulation of UGT1A1 (Figure 6F) and marked decrease of intracellular estrogen products (Figure 6G). It meant the recovery of estrogen metabolism, utilization disorders of estrogens, and estrogen resistance. Meanwhile, Ad-ERα suppressed obviously the proliferation of MDA-MB-231 cells (Figure 6H).

### UGT1A1 Was the Target Gene of miR-452 in TNBC

**Figure 7:**
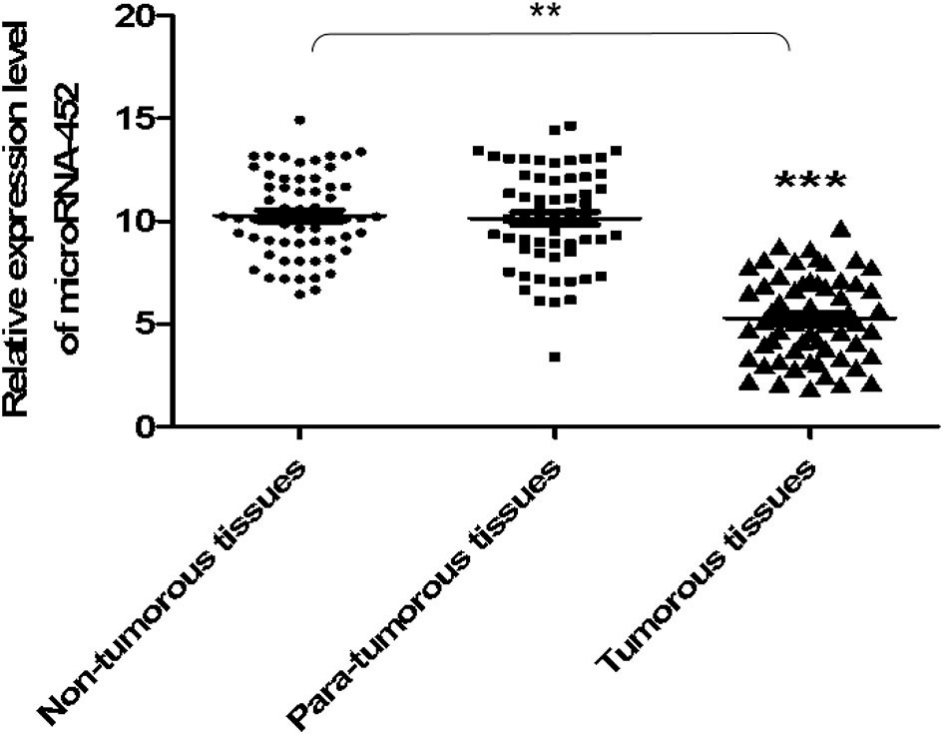
The expression level of miR-452 in tissues of TNBC. The expression level of miR-452 in non-tumorous, para-tumorous, and tumorous tissues of patients with TNBC were detected. The result showed that the relative expression level of miR-452 in tumor was remarkably lower than that of para-tumorous and non-tumorous tissues. However, there was no significant difference between non-tumorous and para-tumorous tissues. **P < 0.01, ***P < 0.001.

At first, the miRNA expression profiles were assessed using next-generation sequencing. Then, 12 differential miRNA expressions were sought out between tumorous and para-tumorous tissues. Ten upregulation miRNAs and two downregulation miRNAs in tumor were found compared with para-tumor tissues (Table 3). Furthermore, miR-452 expressions were tested in tissues of patients with TNBC. The results demonstrated that the relative miR-452 expression level in tumor was remarkably lower than in non-tumorous and para-tumorous tissues. However, no significant difference was found between non-tumorous and para-tumorous tissues (Figure 7).

**Table 3:** Differentially expressed miRNAs in tumor and tumorous tissues (fold-change > 2.0 and *P* < 0.01).

**miRNA**	**Fold-change (tumor/para-tumorous tissues)**	***P*-value**
miR-520b	5.64	0.017
miR-499a	4.91	0.001
miR-122	4.82	0.000
miR-380	4.07	0.045
miR-551a	3.73	0.002
**miR-452**	3.25	0.006
miR-599	2.59	0.015
miR-605	2.56	0.013
miR-372	2.48	0.007
miR-582	2.37	0.008
miR-210	0.45	0.029
miR-208	0.28	0.040

*The meaning of the bold font is the research target in this paper*.

**Figure 8:**
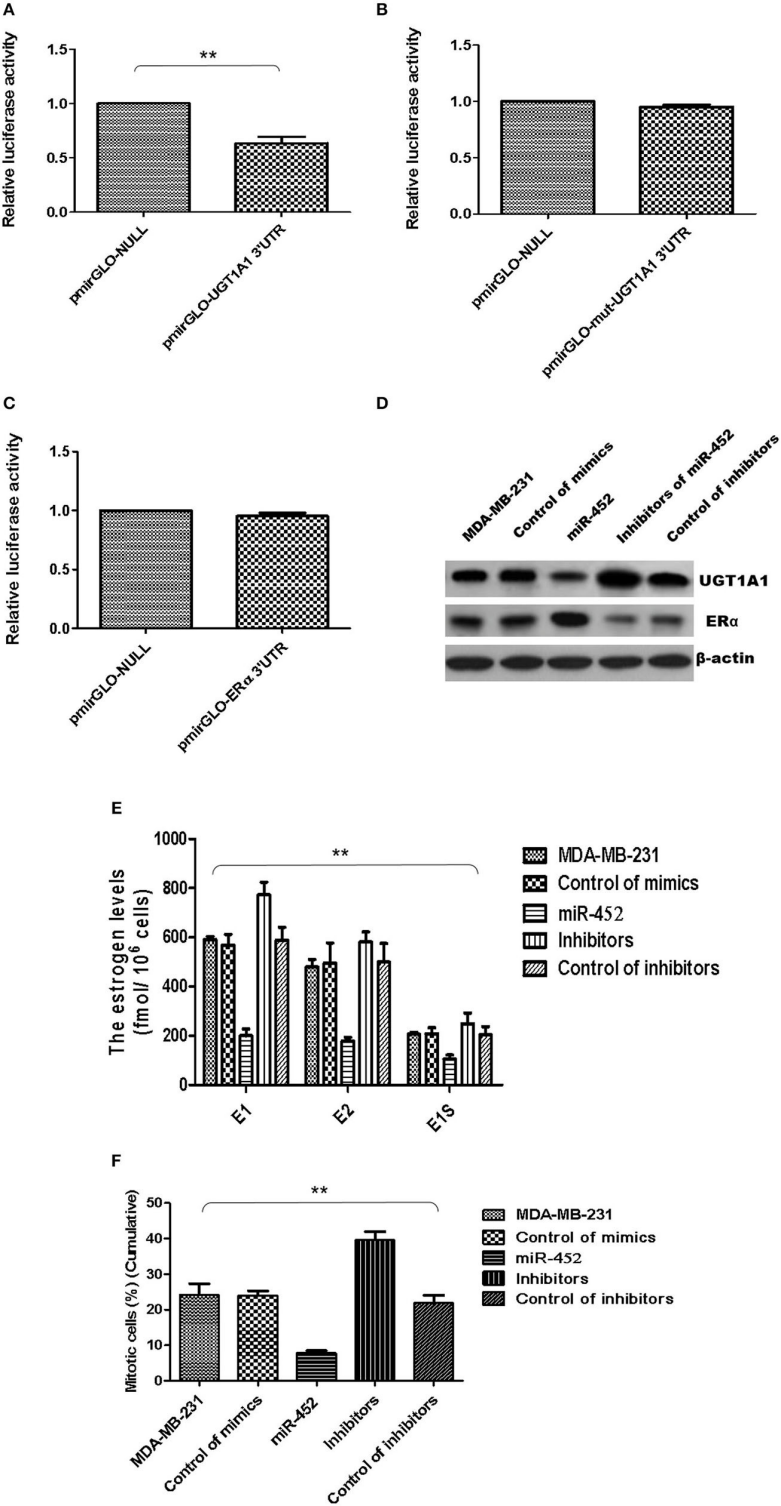
miR-452 inhibited UGT1A1 expression and its bio-functions in TNBC. Further experiments were conducted using luciferase reporter gene system to identify whether miR-452 could straightly bind with 3′UTRs of UGT1A1 mRNA. The data indicated 3′UTR luciferase activities of UGT1A1 significantly downregulated in TNBC treated with miR-452 **(A)**. However, it did not show an obvious change in the group of UGT1A1 mutation **(B)**. Moreover, miR-452 did not bind straight to 3′UTRs of ERα **(C)**. Besides, miR-452 restrained UGT1A1 protein expression level and promoted ERα protein expression in TNBC cell line MDA-MB-231 **(D)**. Moreover, miR-452 inhibitors could promote expression of UGT1A1, but suppress the ERα protein expression level, respectively **(D)**. Furthermore, miR-452 induced a remarkable decrease in intracellular estrogen products in cell line MDA-MB-231 **(E)**. Meanwhile, the inhibitors of miR-452 significantly reduced cell proliferation of MDA-MB-231 **(F)**. **P < 0.01.

Besides, UGT1A1 was predicted as the target gene of miR-452 with Targetscan (version 7.2), an online software. The further identified reporter gene assay was conducted, and recombinant pmirGLO-UGT1A1 was constructed based on 3′UTR sequence of UGT1A1 mRNAs. Our results confirmed that UGT1A1 3′UTR mRNAs could be bound straightly by miR-452 (Figure 8A), but not found in mutation of UGT1A1 (Figure 8B) or ERα (Figure 8C). It is suggested that UGT1A1 was the target gene of miR-452 in TNBC.

Moreover, the UGT1A1 and ERα expressions in protein levels were conducted in MDA-MB-231 cell line. Then the data verified that miR-452 inhibited expression of UGT1A1 and promoted ERα protein expression (Figure 8D). Moreover, miR-452 inhibitors could promote expression of UGT1A1 but suppress the ERα protein expression level, respectively (Figure 8D). In summary, UGT1A1 was the target gene of miR-452, and miR-452 could subsequently affect the expression of its interaction protein ERα.

Furthermore, miR-452 induced a remarkable decrease of intracellular estrogen products in cell line MDA-MB-231 (Figure 8E). It meant the recovery of estrogen metabolism, utilization disorders of estrogens, and estrogen resistance. Meanwhile, the inhibitors of miR-452 reduced remarkably the proliferation of MDA-MB-231 (Figure 8F).

### miR-452 Antagomir Restrained Xenograft of TNBC

**Figure 9:**
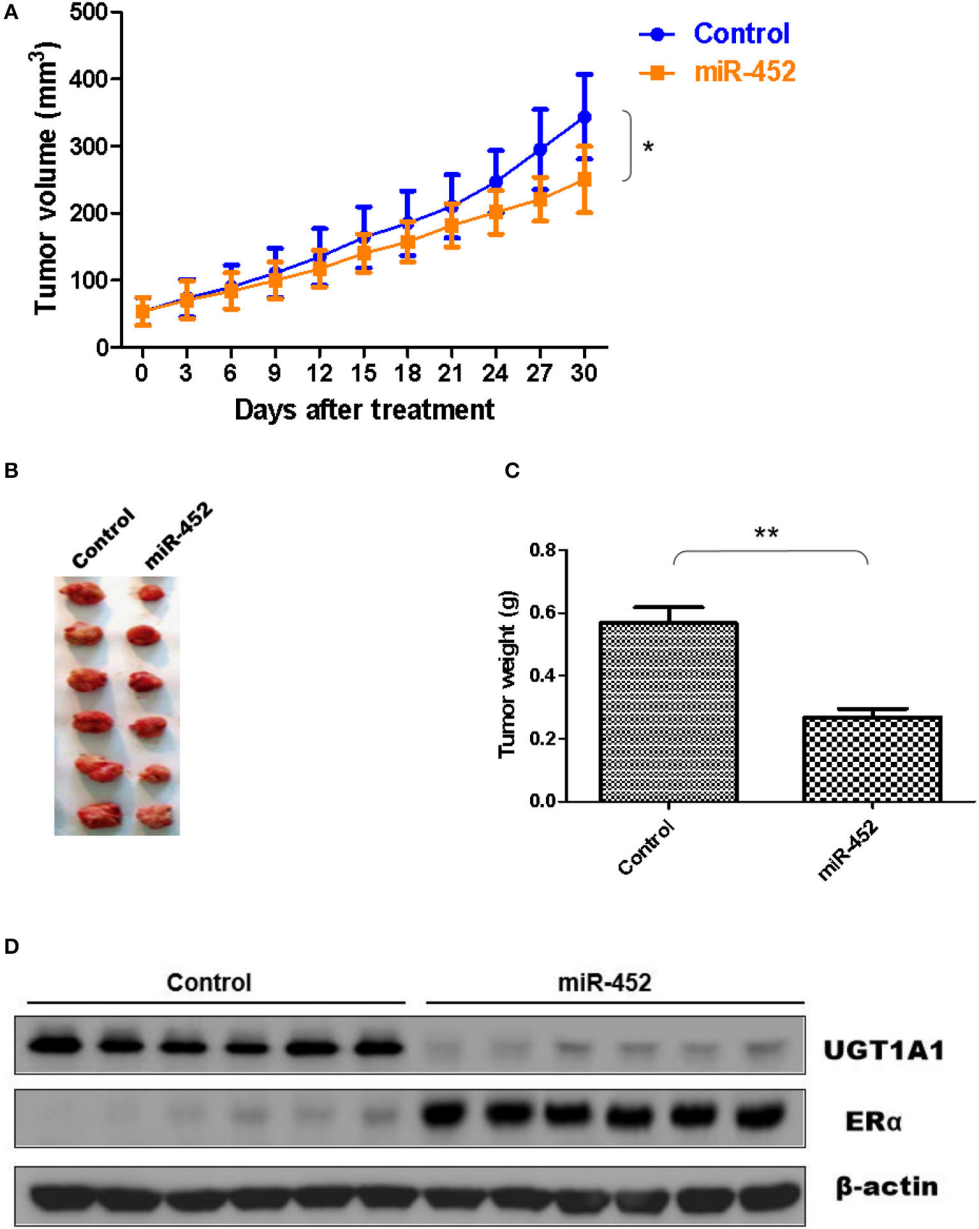
miR-452 antagomir suppressed TNBC xenograft. To detect anti-tumor activity induced by miR-452 antagomir *in vivo*, human TNBC xenografts were established with MDA-MB-231 cell line. The xenografts of control groups grew rapidly, but in the experiment group, which was treated with antagomir of miR-452, they grew slowly *in vivo* **(A)**. Moreover, the weight of tumors in the experiment mice (treated with antagomir of miR-452) was significantly less than that of the control group **(B,C)**. At the time of end, the average tumor volume of control mice was much larger than that of the experiment group, and the difference between the two groups had statistical significance **(C)**. As we can see, the results revealed that compared with the control group, treatment with antagomir of miR-452 downregulated the expression of UGT1A1 with statistical significance and promoted protein expression of ERα in xenografts **(D)**. *P < 0.05, **P < 0.01.

Furthermore, the antagomir of miR-452 was used to examine its anti-tumor effect *in vivo*, and xenografts were constructed using human TNBC cell line MDA-MB-231. The results proved miR-452 antagomir induced slow growth of xenografts *in vivo* compared with rapid growth tumor of control group (Figure 9A). Meanwhile, tumor weights in mice treated with miR-452 antagomir were less notable than that in control (Figures 9B,C). However, average tumor volumes of control mice were much larger obviously than rats treated with miR-452 antagomir (Figure 9C). Moreover, in xenografts, miR-452 antagomir treatment prominently induced the downregulation of UGT1A1 and upregulation of ERα (Figure 9D).

## Discussion

One of the main physiological functions of estrogen is to maintain the normal structure and function of the mammary lobules. As a hormone-dependent tumor, estrogen also provides the vital effect on BC development. Prospective studies suggest that plasma estrogen levels in postmenopausal women are significantly positively associated with the risk for BC while they are not receiving hormone replacement therapy (4, 33, 34). In addition, another large-scale prospective study confirms that endogenous estrogen is related to risk of BC in premenopausal women, and women undergoing estrogen replacement therapy after menopause and exogenous estrogen supplementation increase the risk for BC (35). On the one hand, estrogen acts as a cancer promoter by increasing mitosis of mammary duct epithelial cells. The mechanism may be that as the initiator of cancerization, estrogen metabolites can bind to DNA, then directly cause DNA damage and genetic mutations (36).

Estrogen plays an important role in BC development and occurrence, whereas endocrine therapy can inhibit tumor growth by decreasing the estrogen level in the body or reducing the effect of estrogen. Endocrine therapy is currently recognized as an effective treatment for BC (~67% of BC patients). Although its efficacy is significant, it only targets ER/PR-positive tumors (37). However, TNBC cells lack hormone receptors, so they are not effective for endocrine therapy.

At present, the mechanism of action of anti-estrogen drugs and their resistance are still not fully clarified. In fact, there are two forms of anti-estrogen resistance: neonatal resistance and acquired resistance (38). Lack of expression of ER causes initial resistance, which is a well-known mechanism. However, ineffective anti-estrogen appears to be the main acquired resistance phenotype. There are two subtypes of ER (ERα and ERβ), and ERα is the main determinant of breast epithelial cell development and proliferation (39). The prediction for the response to anti-estrogen therapy only requires detection of ERα expression (38).

The important biological properties of estrogen are applied by binding to specific receptors of target cells. It has been confirmed that special estrogen receptors are hidden in breast epithelial cells. Nearly 50% of intraluminal breast epithelial cells are not actually considered to express ER or have a low level of ER expression (33). Different parts of ER may play different roles in inducing luminal BC. The ER expression level, whether or not estrogen dependent, has a great effect on cell functions (33, 39). Moreover, the role of ER is actually bidirectional. It can promote cell growth and expansion of breast in young mice, but it can also inhibit the growth of breast cells during pregnancy. Similarly, relevant research results suggest that ERα has a very important influence in BC development (33, 39). The occurrence and development of BC (especially TNBC) are closely related to ERα expression, estrogen metabolism, and estrogen resistance (39).

The research conclusion of Stéphanie et al. was consistent with the previous research results of our group (5). The previous research of this research group also found that, in fact, TNBC expressed ER, but its expression level was low and its expression state was abnormal. We found ERα expressed on the surface of TNBC cells at low expression levels.

DXME plays a vital effect in drug response and carcinogenesis. The earlier results of our research group clearly suggested significant different expression of UGT (UDP-glucuronosyltransferases) family in BC (21). UGTs can catalyze the glucuronidation process, which is closely associated with estrogen metabolism. Among them, UGT1A1 expression is related to TNBC treatment strategy and prognosis (21). Therefore, UGT1A1 may be a potential target for research on the pathogenesis of TNBC. Previous studies have shown that UGT1A1-catalyzed glucuronidation is related to the metabolism and detoxification process of estrogen (40, 41). Recently, studies have shown that UGT is closely related to estrogen metabolism. UGT participates in inactivation of E1, E2, and their 2-, 4-hydroxylated derivatives. The cell line of TNBC treated with E2 can produce dose dependence of UGT1A10 mRNA upregulation, whereas anti-estrogen drug ICI 182780 blocks the UGT1A10 mRNA expression level (42). The aforementioned data indicate that ER may medicate the UGT1A10 mRNA expression regulation. Also, other estrogen compounds can also stimulate UGT1A10 mRNA expression, such as Gen (genistein) and PPT (propylpyrazole triol). UGT1A protein expression and enzyme activity were upregulated when TNBC cells were exposed to 0.1 nM E2. The results showed that E2-induced UGT1A expression may be mediated by ER, and UGT may play an important role in estrogen utilization and clearance process of estrogen by regulating estrogen metabolism, thereby further promoting E2 signaling in TNBC cells (42).

In this study, we first assessed the differential mRNA expression profiles between tumorous and para-tumorous tissues. Then, the upregulation of UGT1A1 mRNA was found in the tumor of patients with TNBC. Our results further indicated a high UGT1A1 expression level in TNBC tumor tissues. UGT1A1 was shown in the nucleus, cytoplasm, and cell surface of para-tumor tissue at a low expression level. Moreover, the expression of UGT1A1 in tissues of patients with TNBC was much more than the matched para-tumorous tissues. Meanwhile, there is a low expression level of ERα with the abnormal expression status in TNBC cells; ERα was present on the nucleus, cytoplasm, and cell surface of normal mammary glands at a moderate expression level.

Our previous work predicted through bioinformatics that there was a glycosylation modification site at the 10th amino acid position after ERα translation, and the modification method was N-acetylglucosamine (O-GlcNAc) glycosylation modification. O-GalNAc glycosylation modification plays a vital biological effect in many life activities including individual development, tumorigenesis, or cell adhesion (43). We found that the glycosylation modification site of ERα expressed in TNBC cells is different from that of normal mammary epithelial cells based on mass spectrometry. The glycosylation modification site at the 10th amino acid site is UDP-GlcNAc glycosylation modification in TNBC cells.

However, what is the relationship between the abnormal glycosylation of ERα on the surface of TNBC cells and the occurrence, development of TNBC, or estrogen resistance? What is the mechanism? In response to these scientific issues, we first conducted a preliminary exploratory mechanism study. Therefore, we found that E2 significantly promoted mitotic entry of TNBC cells. In summary, these results sustained powerfully the estrogen as a positive factor in the proliferation of TNBC cells at onset of mitosis through accentuating the expression and enzyme activity of UGT1A1. Besides, the interaction between UGT1A1 and ERα affected the expression level of each other as well as the UGT enzyme activity and proliferation of TNBC cells. Interestingly, UGT1A1 induced production of intracellular estrogens and TNBC proliferation. It meant the suppression of estrogen metabolism, utilization disorders of estrogens, and estrogen resistance. However, overexpression of ERα was induced in cell line MDA-MB-231 that caused downregulation of UGT1A1 and a marked decrease of intracellular estrogen products, and then reduced TNBC proliferation. It meant the recovery of estrogen metabolism, utilization disorders of estrogens, and estrogen resistance.

UGT1A1 participates in the estrogen metabolism process, and it exerts the activity of glucuronosyltransferase to make uridine diphosphate combine with estrogen to be discharged out of the cell, and finally be excreted through the urine. In addition, UGT1A1 also has glycosyltransferase activity and can act on glycosylation modification of ERα at the same time. The possible mechanism is that the normal O-GlcNAc glycosylation modification of ERα requires UGT1A1 to perform the first stage of UDP-GlcNAc glycosylation modification before proceeding. However, owing to the enhanced expression of UGT1A1 in TNBC cells, ERα only completed the first stage, namely UDP-GlcNAc glycosylation, and then stopped immediately, which eventually led to abnormal glycosylation of ERα (44). Our previous work also confirmed that UGT1A1 was overexpressed in TNBC cells and induced downregulation of ERα expression, then resulted in estrogen metabolism utilization disorders and estrogen resistance, thereby further affected the biological function of TNBC cells.

Many studies have shown that microRNAs were associated with pathogenesis in different forms of tumors (45–48). Therefore, further comparison and analysis of the microRNA expression profiles among TNBC patients' tumor tissues and their control tissues adjacent to cancer, or other types of breast cancer tumor specimens and normal control breast tissues were conducted. It was found that there was a significant difference in expression of miR-452. We conducted a bioinformatics prediction study and found that UGT1A1 may be the regulated aim of miR-452. Further experiments confirmed UGT1A1 3′UTRs mRNA could be bound straightly by miR-452 and miR-452 inhibited UGT1A1 expression in TNBC cell line. Moreover, UGT1A1 3′UTR mRNAs could be bound straightly by miR-452, and protein expression of UGT1A1 and ERα were all regulated in a stepwise manner. Besides, our results demonstrated that the miR-452 antagomir induced slow growth of xenografts. In xenografts, miR-452 antagomir treatment induced prominently the downregulation of UGT1A1 and upregulation of ERα.

Multiple studies have shown that miR-452 was associated with the regulation of various tumor biological functions. Studies have confirmed that miR-452 could inhibit proliferation and metastasis in lung cancer and prostate cancer cells (49). Studies have also confirmed that miR-452 exerts growth inhibitory effects on T-cell acute lymphoblastic leukemia. miR-452 was epigenetically silent and targets BMI-1 to exert growth inhibitory activity in T-ALL. The restoration of miR-452 expression may provide hope and treatment strategies for the treatment of this malignant tumor (50–54).

In a word, our work validated that miR-452 suppressed TNBC development related to estrogen metabolism and utilization disorders or estrogen resistance by aiming the UGT1A1 expression and its interaction with ERα. So far, we screened the important target gene UGT1A1 in fate of TNBC through theoretical speculation and experimental verification (55). We expect to select UGT1A1 as the target for the treatment of TNBC, and miR-452 as the core drug to inhibit the expression of UGT1A1 by reversing the abnormal glycosylation modified ERα in TNBC cells, to improve the TNBC estrogen metabolism utilization process and estrogen resistance, and thus to achieve the therapeutic effect on TNBC. It is suggested that the aim at glycosylation modification of ERα and estrogen resistance associated with UGT1A1 by promoting expression of miR-452 was a potential tactic for treatment of TNBC.

## Data Availability Statement

The raw data supporting the conclusions of this article will be made available by the authors, without undue reservation.

## Ethics Statement

The studies involving human participants were reviewed and approved by Ethics Committee of Peking Union Medical College Hospital, Peking Union Medical College. The patients/participants provided their written informed consent to participate in this study. The animal study was reviewed and approved by the ethics committee of Peking Union Medical College, Chinese Academy of Medical Sciences.

## Author Contributions

YLi, JZ, and QS designed the study. YZ, FM, SS, BZ, and YaX collected the data and performed data analysis. YLi, YLin, XZ, XC, JZ, and QS wrote the article. YiX, CC, JZ, and QS revised the article. All authors read and proofread the article.

## Conflict of Interest

The authors declare that the research was conducted in the absence of any commercial or financial relationships that could be construed as a potential conflict of interest.
